# My brain knows numbers! - an ERP study of preschoolers’ numerical knowledge

**DOI:** 10.3389/fpsyg.2013.00716

**Published:** 2013-10-21

**Authors:** Tamar Ben-Shalom, Andrea Berger, Avishai Henik

**Affiliations:** Department of Psychology and Zlotowski Center for Neuroscience, Ben-Gurion University of the NegevBeer Sheva, Israel

**Keywords:** automatic numerical processing, size congruity effect, ERP, distance effect, brain development

## Abstract

This study investigated brain activity in numerical processing at early stages of development. Brain activity of preschoolers was measured while they performed a numerical Stroop task. Participants were asked to decide which of two digits was numerically or physically larger. Behavioral distance and size congruity effects (SiCEs) were found. However, a reverse facilitation was observed, where responses to neutral trials were faster than to congruent ones. The event-related potentials data showed the expected distance effect at occipitoparietal scalp areas. Moreover, conflict was related to effects both at frontal and parietal scalp areas. In addition, there was a difference between the timing of the interference compared to the facilitation components in the SiCE. In parietal scalp areas, facilitation was significant in an early time window and interference was significant at a later time window. This is consistent with the idea that facilitation and interference are separate processes. Our findings indicate that children as young as 5–6 years old can automatically process the numerical meaning of numerals. In addition, our findings are consistent with the idea that, children might use both frontal and parietal areas in order to process irrelevant numerical information.

## INTRODUCTION

### BEHAVIORAL EFFECTS OF NUMERICAL PROCESSING

Our purpose was to investigate how children process numerical values of numerals and how this processing is reflected in their brain activity. Therefore we chose to focus on behavioral effects that indicate automatic processing of numerals. In the study of numerical processing of numerals, two common effects are usually investigated and reported: the *distance effect* (DE) and the *size congruity effect* (SiCE). The DE can be measured when two numerals differ in their numerical value. It was found that subjects are quicker to compare two numerals that are farther apart (e.g., 2 8) than closer ones (e.g., 2 3). This effect was first reported by Moyer and Landauer (1967). The DE was replicated in many studies since then (Henik and Tzelgov, 1982; Dehaene et al., 1990; Tzelgov et al., 1992b). The DE is considered to be an indication for the existence of an analogical mental number line that contains representations of numerals. Representations of close numerals (e.g., 1 2) appear closer on the mental number line than representations of farther apart numerals (e.g., 2 8). Each numeral has its own representative space that overlaps that of the neighboring numerals, and as a result, comparisons are slower for small numerical distances than for large numerical distances.

The SiCE is considered to be evidence for automatic numerical processing. Henik and Tzelgov (1982) were the first to test subjects using the numerical Stroop paradigm. They found that subjects were quicker judging physical sizes when they were congruent to numerical values of the numerals presented (e.g., 3 5). Subjects were slowest when physical sizes and numerical values were incongruent (e.g., 3 5). Henik and Tzelgov considered this as evidence that the numerical dimension is processed in a non-intentional and automatic manner (Henik and Tzelgov, 1982; Tzelgov et al., 1992b; Rubinsten et al., 2002). Also, by adding a neutral stimulus to the task (e.g., 3 3) it enabled dividing the congruity effect into two components – the *interference* component (incongruent minus neutral trial reaction times, RTs) and *facilitation* component (neutral minus congruent trial RTs). This way, one can examine whether the congruity effect is mostly created by the interference of the irrelevant dimension (the numerical dimension, which the subjects were asked to ignore), or by the facilitation of the irrelevant dimension.

Studies that explored the components of the Stroop effect (i.e., facilitation and interference) tried to dissociate these two components in order to understand what they represent (Posner, 1978; MacLeod and Dunbar, 1988; Tzelgov et al., 1992a; Lindsay and Jacoby, 1994; Szũcs and Soltész, 2007, 2008). Rubinsten and Henik (2006) suggested that the facilitatory component is supposed to involve processes that are more automatic because they are less subjected to strategic control (e.g., see Tzelgov et al., 1992a). Posner (1978) also suggested that facilitation is an indicator of automaticity, whereas interference might reflect attentional processing.

### THE DEVELOPMENT OF BEHAVIORAL EFFECTS OF NUMERICAL PROCESSING

Studies that investigated the *development* of the DE and the SiCE and its components (interference and facilitation) found that young children (preschoolers) already showed some of these effects. The DE was found among 5- to 8-year-olds (Sekular and Mierkiewicz, 1977; Duncan and McFarland, 1980; Temple and Posner, 1998; Rubinsten et al., 2002; De Smedt et al., 2009; Holloway and Ansari, 2009). The results indicated that 5-year-olds already have a mental number line and can use it when necessary. As for the SiCE, this effect was considered to appear only among first or second graders (Girelli et al., 2000; Rubinsten et al., 2002; Mussolin and Noël, 2007, 2008). Rubinsten et al. (2002) studied the development of the components of the SiCE: interference and facilitation. They found that children at the beginning of first grade did not present either of these two components in the physical task (when the numerical dimension was irrelevant). However, children at the end of first grade presented a significant interference effect but not facilitation in the physical task (when the numerical value was irrelevant). This finding might indicate that the facilitation component is more automatic than the interference component and hence appears later among young children, when the automatic numerical processing is more stable and even automatic in its nature. To our knowledge, up until now only one study by Zhou et al. (2007) demonstrated the SiCE at younger ages – among preschoolers (5–6 years old). They related their results to cultural differences between Chinese children and the population of children that have been studied to date.

**FIGURE 1:**
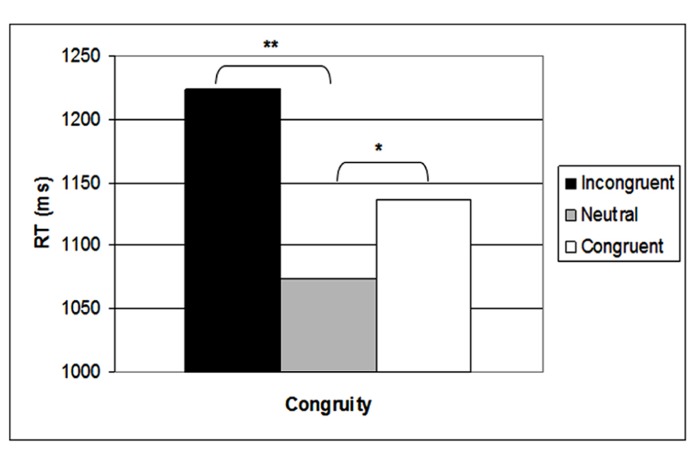
**Behavioral SiCE in the physical task.** *p < 0.05 (two-tailed), **p < 0.01 (two-tailed).

In a previous behavioral study (Ben Shalom et al., unpublished), we found a significant SiCE among preschoolers (5–6 years old), which showed significant interference and a significant reverse facilitation (see **Figure 1** for similar results). We related this pattern of results to non-mature numerical processing of numerals. These children could automatically relate to the incongruity between physical size and numerical dimensions, and therefore presented an interference effect, but they could not automatically relate to the congruity between the physical and numerical dimensions, and therefore presented a reversed facilitation, meaning that the neutral trials were the easiest for them to judge. After receiving these novel results, we were interested in examining the brain mechanisms behind the numerical processing of these young children.

### BRAIN ACTIVITY OF NUMERICAL PROCESSING AND ITS DEVELOPMENT

Neuroimaging studies found that specific parietal areas are activated during numerical processing tasks, and more specifically, the intraparietal sulcus (IPS; Dehaene et al., 2003, 2004; Pinel et al., 2004). This area was found to be modulated by the numerical difference between numerals (i.e., DE; Dehaene et al., 2003; Pinel et al., 2004) and by the incongruity between numerical and physical dimensions (i.e., SiCE; Cohen Kadosh et al., 2007).

Several studies have examined the development of these brain mechanisms of numerical processing. Using functional magnetic resonance imaging (fMRI), Ansari et al. (2005) examined developmental differences in functional neuroanatomy of symbolic number processing. Their results indicated that children’s numerical DE was found significant in frontal areas, whereas adults showed this effect in parietal areas. They concluded that this might be an indication for ontogenetic shift throughout development, toward greater parietal engagement in symbolic numerical comparison. Kaufmann et al. (2005) investigated the numerical Stroop effect in children compared to adults. They found that in adult brains, the congruity effect in the numerical Stroop task was seen in the dorsolateral prefrontal cortex and anterior cingulated cortex, and was related to attentional control. Additionally, a larger distance between numerosities resulted in a greater activation in bilateral parietal areas, including the IPS. Kaufmann et al. (2006) also found that the same task activated different brain areas in 9-year-old children. Brain areas that were activated when there was a large numerical distance were frontal but not parietal for the children group. Also, when the numerical value was irrelevant (in the physical task), frontal areas were more activated when comparing the incongruent stimulus activation to the neutral one.

Event-related potential (ERP) studies also investigated the course of the development of the DE and SiCE. Dehaene (1996) found voltage differences associated with numerical distance in a comparison of digits task among adult subjects. This effect was found in the time window of 174–230 ms after stimulus presentation, in electrodes of the occipito–parieto junction. Temple and Posner (1998) examined the development of this brain activity in relation to numerical distance in children. Their study replicated Dehaene’s results regarding adults’ ERP topography, although they found the effect in an earlier time window (124–234 ms after stimulus presentation). They also revealed the same voltage differences for numerical distance were found among 5-year-old children in a numeral comparison task, although they were slightly delayed compared to adults (around 50 ms after the adults’ window). Although it should be mentioned, that in this study, the small and large numerical distance conditions were not perceptually balanced. Hence, ERPs may have been affected by perceptual effects.

Szũcs et al., (2007) also examined the development of brain activity during numerical processing. They examine adults and 9- to 11-year-old children using the numerical Stroop task. They replicated results of previous studies finding that both children and adults demonstrated significant voltage differences for numerical distance, between 140 and 320 ms after stimulus presentation and mostly over right parietal electrodes. These findings suggested that children and adults can access the representation of the number line at a similar speed. Looking into brain activity of interference and facilitation components in physical comparisons, Szũcs et al., (2007) also found different brain activity patterns for children compared to adults. Specifically, two wave components – the P300 and lateralized readiness potential (LRP) – were found to be different between those age groups. The interference effect of the Stroop task was more related to the LRP wave (i.e., response conflict) component for children compared to adults. In their opinion, this result indicated that children’s slower response in a numerical comparison task was due to an unorganized behavioral response. They concluded that different cognitive processes underlie children’s performance in the numerical Stroop task due to non-matured executive function that is required to carry out this task. Soltész et al. (2011b) examined the SiCE in physical judgment among children in grades 1–3. In their article, they stated that they expected stronger interference effects in younger children due to the relatively immature functioning in the prefrontal cortex, but that interference might weaken in favor of facilitation in older children as number processing became more and more automatic. This also supports our hypothesis about the pattern of interference vs. facilitation that we expected to find among 5- to 6-year-old children, as we found in a previous behavioral study.

## THE PRESENT STUDY

The purpose of our study was to examine the development of brain mechanisms of DE and SiCE in children of younger ages than were studied so far, that is, among 5- to 6-year-old children, by using an ERP method. According to previous findings, we hypothesized the following:

• Preschoolers would show automatic numerical processing by expressing a significant behavioral DE in the numerical task and SiCE in the physical task.
• Preschoolers would show significant voltage differences to numerical distance according to previous studies (Dehaene, 1996; Temple and Posner, 1998; Szũcs et al., 2007), in the same time window (124–234 ms after stimulus presentation), in electrodes of the occipito–parieto junction.
• If kindergartners showed a significant behavioral SiCE, the ERP topography of this effect would be more frontal than parietal, according to previous studies (Ansari et al., 2005; Kaufmann et al., 2006; Szũcs et al., 2007; Szũcs and Soltész, 2008; Soltész et al., 2011a). Regarding time window, we did not have a specific time window from previous literature because our study is the first one that we are familiar with, that investigated ERP patterns of the SiCE in such young ages.

### METHOD

#### Participants

Seventeen preschoolers – eight males and nine females – aged 5–6 years old (average of 5.5 years old) without any learning or developmental disabilities (based on parental reports) were examined. Children’s parents were given payment for their participation. Adult students were given course credit. Parental consent was obtained for the children.

#### Procedure

Families were contacted through their children’s kindergarten. For those families who agreed to participate, a home visit was scheduled to assess the child’s IQ and basic numerical abilities. We verified in a home visit that each child knew how to count up to 10 and recognized the numerals 1–9. We also administered the colored RAVEN IQ test in order to measure the child’s IQ level. Subsequently, a lab visit was scheduled in which the child performed the numerical Stroop task while his/her brain activity was measured.

The average time for this meeting was 1 h. The task took 20 min (on average) for each child. The order of the tasks was counterbalanced: half of the subjects were tested first on the numerical judgment task and the other half were tested first on the physical judgment task. At the beginning of each task, the participant preformed 12 practice trials where positive feedback for correct answers was given. The experimental blocks themselves did not include any feedback.

At the end of each meeting, parents were given payment for their child’s participation. The research was approved by the Israeli Ministry of Education and by the Helsinki Committee.

#### Stimuli

Two digits appeared in each trial at the center of a computer screen. The distance between the two digits was 10 mm. A typical trial started with a fixation point presented for 300 ms, followed by the two digits that remained in view until the participant pressed one of the computer buttons to indicate which digit was larger. In two separate blocks participants were asked to compare the numerical values of the two digits or their physical sizes. In the numerical block, the two digits differed only in their numerical size and not in their physical size. This was done in order to reduce the amount of trials required for the children’s experiment. In the physical block the two digits differed in their numerical and physical sizes. There were only two possible physical sizes: the size of the larger digit was 13 mm and the smaller one was 10 mm.

The stimuli in each block were created using the digits 1, 2, 3, 4, 6, 7, 8, and 9. From these numerals we created two numerical distances: 1 (the pairs: 1–2, 3–4, 6–7, 8–9) and 5 (the pairs: 1–6, 2–7, 3–8, 4–9). Thus, there were eight different stimuli pairs. The pairs could appear with the larger number on the right or with the larger number on the left, allowing for 16 pairs. In the numerical block these stimuli were repeated four times for a total of 64 stimuli. In the physical block, the 16 pairs of stimuli could have each digit appear in two different physical sizes, allowing for 32 different stimuli. The congruent stimuli (e.g., 3 8) were repeated as necessary to create 32 congruent stimuli; the same process was carried out to create 32 incongruent stimuli (e.g., 3 8). The 32 neutral stimuli were created using a pair of two digits that differed in the physical dimension but not in the numerical dimension (e.g., 2 2). Thus, the physical block contained 96 different stimuli.

***EEG recording and analysis.*** E-Prime 2.0 (Psychology Software Tools, Pittsburgh, PA) was used for stimuli presentation and behavioral data collection. An electroencephalogram (EEG) was recorded from 128 scalp sites using the Electrical Geodesics, Inc. (EGI) Geodesic Sensor Net and system (Eugene, OR; Tucker, 1993). Electrode impedances were kept below 40 kΩ, an acceptable level for this system (Ferree et al., 2001). Data was processed using a 0.1–100 Hz bandpass filter. Signals were collected at 250 samples per second and digitized with a 24-bit A/D converter. EEG data from trials that were included in the behavioral analysis were processed in Netstation, v4.3 (Electrical Geodesics, Eugene, OR).

Using the Netstation program, continuous EEG data were filtered with a 0.3 Hz high pass and 47 Hz low pass (following Szũcs et al., 2007). The data was then segmented into trials time-locked to the presentation of the stimulus. The length of the segmentation included 100 ms before stimulus presentation and 1,200 ms afterward. Resulting segments were subjected to an automatic bad-channel-, eye blink-, or movement-detection procedure, followed by manual verification. This procedure marks channels as bad if they have a max–min difference higher than 200 μV. It also marks segments with a difference higher than 140 or 55 μV as containing an eye blink or an eye movement, respectively. Segments containing 10 or more bad channels, or those in which any eye activity was detected, were discarded. The minimum number of trials remaining per condition was 25. Before averaging, each trial was re-referenced with the PARE (polar-corrected average reference) re-reference technique for all of the sensors at each time point. Finally, after averaging the trials, subsets were baseline-corrected to 100-ms pre-stimulus presentation and averaged into a grand average of all subjects. Analysis was guided by previous findings of Dehaene (1996); Temple and Posner (1998), and Szũcs et al., (2007), as well as by preliminary visual inspection of the grand-averaged data, using for each effect the difference wave between the conditions. After statistical extraction of the average means for each subject for each condition and time-window, this data was analyzed using a repeated-measures analysis of variance (ANOVA).

Data were analyzed by using repeated-measures ANOVAs for each time window, with numerical distance (1 and 5) and congruity (incongruent, neutral, and congruent) as the within subjects variable. The mean amplitude for the channel group was extracted for each time window for each child in each condition.

The scalp ERP topography that was found for the DE is not fully comparable with the classic location of parietal activity seen in numerical tasks in previous studies (Dehaene, 1996; Temple and Posner, 1998; Szũcs et al., 2007). Therefore, we used preliminary inspection of the difference voltage between the conditions in order to fully capture ERP topography and the time window of the effect. Finally, we used the time window of 284–380 ms after stimulus presentation for the DE, at the ERP topography of the bilateral occipito-parietal area, placing a group of 10 electrodes between P3, P4, O1, and O2 of the 10–20 system. As for the SiCE, visual inspection of the results revealed that children showed voltage differences to congruity conditions in frontal and parietal areas. We again used preliminary inspection of the difference voltage between the conditions. We used three time windows and two ERP topographies: (a) 370–440 ms after stimulus presentation at medial frontal area, placing a group of five electrodes between FZ and CZ of the 10–20 system and (b) 600–750 ms and 810–1190 ms after stimulus presentation at right parietal area, placing a group of five electrodes around P4 of the 10–20 system electrodes.

## RESULTS

### BEHAVIORAL DATA

Mean RTs were calculated for correct responses. RTs were analyzed as the depended variable. We will divide our results into two sections: DE and SiCE in each task. In the numerical task, the numerical distance was the between subjects variable. In the physical block, the congruity effect was the between subjects variable.

#### DE in the numerical task

A significant main effect was found for the numerical distance between numerals [*F*(1,16) = 7.42, MSE = 226,780, *p* < 0.01]. RTs for numerical distance 1 (2,084 ms) were slower than RTs for numerical distance 5 (1,920 ms).

#### SiCE in the physical task

A significant main effect of congruity was found [*F*(2,32) = 17.83, MSE = 97,019, *p* < 0.001]. Planned comparisons showed that incongruent trials were significantly slower than congruent trials [*F*(1,16) = 19.9, MSE = 3,253, *p* < 0.001], and congruent trials were significantly slower than neutral trials [*F*(1,16) = 5.82, MSE = 5,822, *p* < 0.05]. This created a SiCE with a reverse facilitation (see **Figure 1**; incongruent RT = 1,224 ms, congruent RT = 1,136 ms, neutral RT = 1,073 ms). No significant effect was found for numerical distance.

### EEG DATA

Similar to behavioral results, we will divide our results into two sections: DE and SiCE. We found a significant DE, and interference and facilitation effects. As can be seen in **Table 1**, the facilitation appeared to be significant earlier than the interference effect was.

**Table 1:** SiCE effect in frontal and parietal areas.

Localization	Time window (ms)	Effect	*F*	MSE	*p*-Value
Medial–frontal; five electrodes between FZ and CZ	370–440	Congruity	(2,32) = 3.23	2.96	=0.053 [-1pc]
		Facilitation	NS		
		Interference	(1,16) = 3.23	5.77	<0.05
Right parietal area; five electrodes around P4	600–750	Congruity	(2,32) = 5.95	7.75	<0.01 [-1pc]
		Facilitation	(1,16) = 8.01	11.28	<0.01
		Interference	NS		
	810–1,190	Congruity	(2,32) = 5.95	6.48	<0.01
		Facilitation	NS		
		Interference	(1,16) = 9.86	6.11	<0.01

#### DE in the numerical task

**FIGURE 2:**
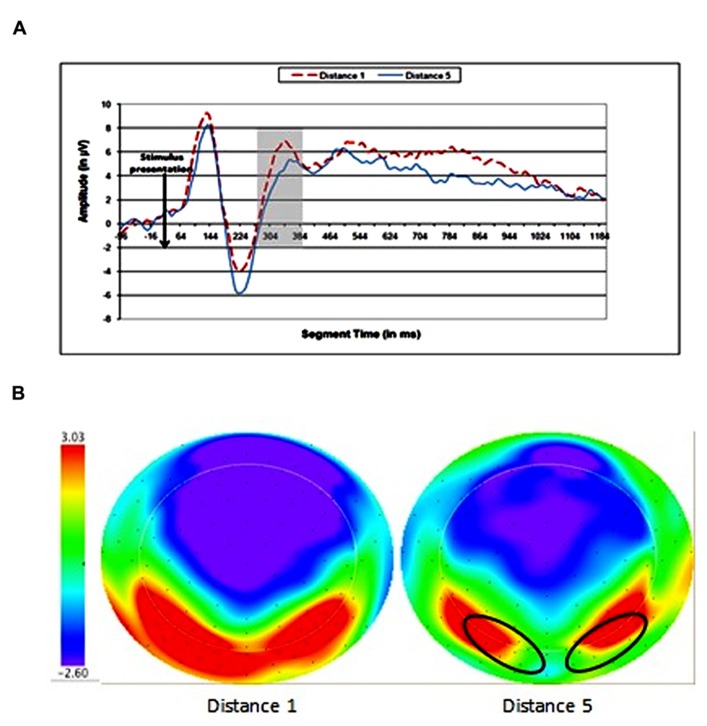
**Electrophysiological results for the numerical distance effect.** **(A)** Wave forms of distances 1 and 5 at occipitoparietal electrode sites. **(B)** Topographic maps of scalp voltages at around 330 ms after stimulus presentation, for distances 1 and 5. A black circle indicates the area of electrodes that were analyzed.

Children showed voltage differences for numerical distance in occipito-parietal areas. The time window that was found significant in the analysis was 284–380 ms after stimulus presentation (see **Figure 2**).

The mean amplitude for the channel group was extracted for each time window for each child in each condition. Data were analyzed by using repeated-measures ANOVAs for each time window, with numerical distance (1 and 5) as the within subjects variable. The ANOVA analysis revealed a significant effect of numerical distance [*F*(1,16) = 4.38, MSE = 6.2, *p* = 0.05] in the time window of 284–380 ms (see **Figure 2**). Other time windows were analyzed and found to be non-significant.

#### SiCE in the physical task

**FIGURE 3:**
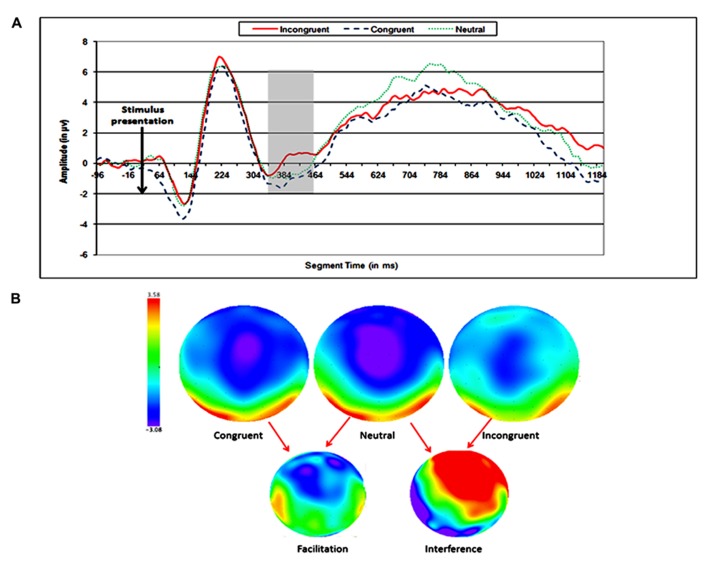
**Electrophysiological results for the earlier SiCE of the physical task.** **(A)** Waveforms of congruent, neutral, and incongruent conditions at medial frontal electrode sites. **(B)** Topographic maps of scalp voltages at around 330 ms after stimulus presentation, for congruent, neutral, incongruent, facilitation (neutral – congruent), and interference (incongruent – neutral). A black circle indicates the area of electrodes that were analyzed.

**FIGURE 4:**
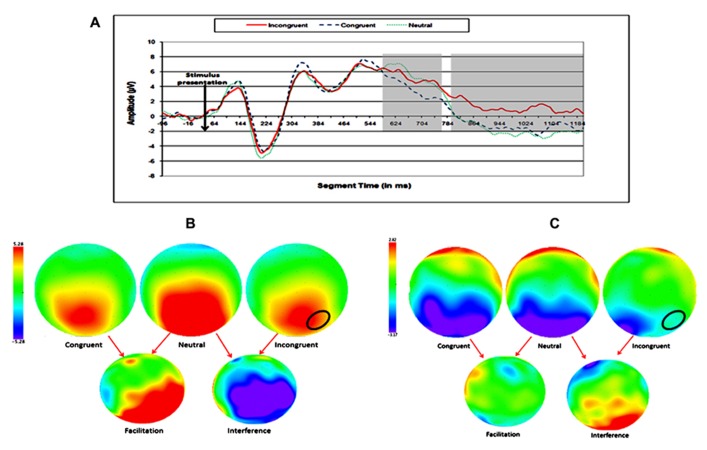
**Electrophysiological results for the later SiCE of the physical task.** **(A)** Waveforms of congruent, neutral, and incongruent conditions at right parietal electrode sites. **(B)** Topographic maps of scalp voltages at around 635 ms after stimulus presentation, for congruent, neutral, incongruent, facilitation (neutral – congruent), and interference (incongruent – neutral). **(C)** Topographic maps of scalp voltages at around 1035 ms after stimulus presentation, for congruent, neutral, incongruent, facilitation (neutral – congruent), and interference (incongruent – neutral). A black circle indicates the area of electrodes that were analyzed.

Two ERP topographies were defined for the analysis of the SiCE, according to the previous finding of Szũcs et al., (2007) and preliminary inspection of the waveforms: (1) medial–frontal and (2) right parietal. No significant effect was found in the left parietal area. One time window was defined for the medial–frontal area and two time windows were defined for the right parietal area (see **Figures 3** and **4**).

## DISCUSSION

Our study examined brain mechanisms of numerical processing among preschoolers. The main results of the study were: (1) a significant behavioral DE and SiCE were found among these children; (2) a significant reverse facilitation was found when the numerical dimension was irrelevant; (3) brain ERP patterns were modulated by numerical distance in occipito-parietal brain areas; and (4) a congruity effect was found in these children in electrodes above frontal and parietal brain areas. We will discuss each result separately.

### BEHAVIORAL DE AND SiCE

Children showed differences in RT according to numerical distance in the numerical task. This result replicates previous findings that showed that children as young as 5 years old can access the mental representation of the number line in a direct comparison task between numerals (Sekular and Mierkiewicz, 1977; Duncan and McFarland, 1980; Temple and Posner, 1998; Rubinsten et al., 2002; De Smedt et al., 2009; Holloway and Ansari, 2009).

In addition, we found a significant behavioral SiCE in the physical task. This is only the second time to our knowledge (except for Zhou et al., 2007) that a SiCE was demonstrated among preschoolers. This finding contradicts several previous studies that found a SiCE only in children at later school ages (Girelli et al., 2000; Rubinsten et al., 2002; Mussolin and Noël, 2007, 2008). A possible explanation could be that today, preschool children are more exposed to numerical stimuli than they were in the past. At least in our country, the current preschool curriculum includes learning of numerals and their association to numerical magnitude. Our behavioral findings indicate that this numerical processing already reached some level of automaticity at this young age.

However, the pattern of the SiCE that the children showed in their RT is still not a fully mature one that characterizes older children and adults in the numerical Stroop task. At preschool age, the children show an inverse facilitation. This means that the RTs to the neutral trials in the physical comparison (e.g., 3 3) were faster than to congruent and incongruent trials. We have already found and reported this pattern in a separate larger sample (Ben Shalom et al., unpublished). Previous findings, such as those of Rubinsten et al. (2002), found that children at the beginning of first grade did not show any facilitation or interference in the physical comparison task. At the end of first grade, children presented an interference component with no facilitation. In our study, the SiCE was significant in the physical task. This suggests that children at this age already have automatic processing of numerical values.

Interestingly, the pattern of the SiCE that we found indicates a reverse facilitation in the physical judgment. RTs to the neutral trials in the physical comparison (e.g., 3 3) were faster than RTs to the congruent and incongruent trials. According to this pattern, the neutral trials were easier to respond to than the congruent and incongruent trials were. One possible explanation for this pattern is the idea that an additional conflict is involved in the numerical Stroop task. Goldfarb and Henik (2007) suggested that Stroop stimuli create two kinds of conflicts – a task conflict and an information conflict. The task conflict is created because there are two tasks that can be applied to the stimulus – naming the color and reading the (irrelevant) word. The information conflict is created because the stimulus carries information along two dimensions – the information provided by the meaning of the word and the information provided by the color of the word. The incongruent pairs create both types of conflicts (information and task). In contrast, the congruent pairs present only the task conflict because the information from both dimensions point in the same direction. The neutral pairs have no conflict at all.

Goldfarb and Henik (2007) reduced cognitive control, and found a reverse facilitation – the neutral trials were faster than congruent trials. Their explanation was that this happened because they revealed the task conflict in the congruent trials. In light of this study it is interesting to find a reverse facilitation in young children and in numerical cognition. The reverse facilitation in our study was not due to manipulation of control. We suggest that this reverse facilitation in kindergarten children is due to premature control ability. Specifically, the children had to switch from comparing the physical sizes to comparing the numerical values (or vice versa). The cognitive ability to switch and manage conflicts is probably not fully developed in these young children. Hence, the congruent condition is still more difficult for them because it contains a conflict (the task conflict). Also, Children in this age group are well trained in differentiating physical sizes, so a comparison between sizes becomes automatic, even when irrelevant, and can be processed fast enough to interfere with or facilitate numerical comparisons. On the other hand, our group of children was not yet well-trained, and thus, they did not automatically respond to the numerical values of numerals. This dimension can create conflict and interference, but it does not facilitate a child’s decision in congruent comparisons. This explanation was also given in Rubinsten et al. (2002). They investigated (among other age groups) first graders in the numerical Stroop task. They found that in this group, in the physical task (when the numerical dimension was irrelevant), size congruity was composed only of the interference component at the beginning of first grade. The facilitation component appeared later, in the older age groups. They concluded that the interference component, which is more automatic in nature, appeared earlier than the facilitation component in the physical task (when numerical values were irrelevant).

Another possible explanation for the reverse facilitation pattern observed in our study relies on the difference in the capability of the children at this age to attend physical sizes, as opposed to numerical sizes. The processing of numerical value, in contrast to physical size, is not enough trained and automatic. Thus, when it is irrelevant, although it creates conflict and interferes, it is not processed fast enough to facilitate a child’s response in congruent trials.

In either case, the reverse facilitation pattern seems to reflect a still relatively immature processing of the numerical dimension of the stimuli, when this dimension is irrelevant to the task. Interestingly, a similar pattern of lack of facilitation or “reverse facilitation” has also been reported (Rubinsten et al., 2002; Rubinsten and Henik, 2006; Ashkenazi et al., 2008).

### OCCIPITO-PARIETAL EFFECTS OF NUMERICAL DISTANCE

We found a significant DE in data gathered from electrodes above the occipito-parietal area in the time window 284–380 ms after stimulus presentation. Our time window for the DE is similar to that reported by Szũcs et al. (2007; i.e., 240–320 ms). Moreover, our topography of the DE (occipito-parietal junction) is very similar to Szũcs et al.’s 2007 ERP topography in grade 3 children (the youngest subjects in their study). Our results partially replicate those of Temple and Posner (1998). However, they found a bi-lateral DE and our time window for this effect is somewhat later. These differences probably result from the fact that in their study the children performed a straightforward numerical comparison, while in our study the children were presented with two competing dimensions.

### FRONTAL AND PARIETAL EFFECTS IN THE SiCE

In our study, the SiCE effect was found in data gathered from electrodes above both frontal and parietal areas. Interestingly, the timing of the congruity effect was earlier in frontal electrodes and later in right parietal electrodes. This result could indicate an earlier detection of the incongruity between the physical and numerical dimension by the frontal lobe. The significant later effect among parietal electrodes could indicate a more detailed processing of the conflict presented by the irrelevant dimension, and the automatic activation of the numerical dimension. Studies have found that children showed more frontal ERP effect when asked to perform numerical comparison (Ansari et al., 2005; Kaufmann et al., 2005, 2006). This frontal ERP effect was related to immature numerical processing that is based more on the executive function system. Our results support this idea, as seen in the early activation of the executive function system (in more frontal areas). However, we found later ERP effect above the right parietal area, which is considered in many studies as the area that is responsible for numerical processing, especially in the numerical Stroop task (Dehaene et al., 2003; Pinel et al., 2004; Cohen Kadosh et al., 2007). Our findings are novel in light of the fact that no research to date has found parietal ERP effect in children at such a young age using the numerical Stroop task.

Another aspect of our results is that they lend support to the research of Szũcs and Soltész (2007, 2008) by differentiating the facilitation and interference components of the SiCE. In our study, as well as in theirs, the facilitation over electrodes above parietal areas appeared earlier than the interference did. In Szũcs and Soltész’s study they relate the facilitation component to the processing stage of the stimuli (regarding the numerical and physical sizes) and the interference to response selection. Our results cannot clearly suggest the same idea, but the time course of parietal activation is similar.

## CONCLUSION

During the last year of kindergarten, children already show an automatic activation of the numerical value of numerals. The pattern of RTs at this age is unique in the physical task (when the numerical value is irrelevant; Ben Shalom et al., unpublished). This pattern of results resembles the pattern of the SiCE of discalculic adults or acalculic patients (Rubinsten et al., 2002; Rubinsten and Henik, 2006; Ashkenazi et al., 2008). In addition, young children show brain activation sensitivity, in ERP effects over frontal and parietal areas, to the numerical distance between numerals and to incongruity between the numerical and physical dimensions. The early activation found in the data gathered from electrodes above frontal areas can be related to conflict management that these children needed to activate in order to process the incongruity between dimensions. The later activation among electrodes above parietal areas could indicate that even more mature networks in the parietal area were activated later and used to process the numerical information. This is the first study to our knowledge that showed this kind of brain activation of the SiCE in children at such young ages (6- to 5-year-olds). Other studies that previously examined the automatic activation of the numerical dimension found this effect only in older-aged children (first–second grade). However, a speculative hypothesis can be made about cohort differences between children who are studied today as opposed to children who were studied in the past. Today in Israel, preschoolers learn the numerals 1–10 and the association between numerals and quantities in a formal way. This can explain the difference between our results and previous results in the literature. However, more research needs to be done in order to expand our and Zhou et al.’s 2007 results regarding preschoolers’ numerical automatic processing in order to fully understand this effect and its relation to individual differences in children’s numerical abilities.

## Conflict of Interest Statement

The authors declare that the research was conducted in the absence of any commercial or financial relationships that could be construed as a potential conflict of interest.
